# Predictors and morphological properties of culprit healed plaques in patients with angina pectoris

**DOI:** 10.1002/clc.23896

**Published:** 2022-09-07

**Authors:** Hongying Yao, Qianyun Guo, Yujing Cheng, Tianyu Zhu, Qian Ma, Yujie Zhou

**Affiliations:** ^1^ Department of Cardiology, Clinical Center for Coronary Heart Disease, Beijing Key Laboratory of Precision Medicine of Coronary Atherosclerotic Disease, Beijing Institute of Heart Lung and Blood Vessel Disease, Beijing Anzhen Hospital Capital Medical University Beijing China; ^2^ Department of Cardiology Peking University International Hospital Beijing China

**Keywords:** angina pectoris, atherosclerosis, healed plaques, optical coherence tomography

## Abstract

**Background:**

Plaque healing may serve a vital function in the natural progression of atherosclerotic disease. This study sought to investigate predictors and morphological characteristics of healed plaque (HP) among angina pectoris (AP) patients.

**Methods:**

Patients who presented with AP and received preintervention optical coherence tomography (OCT) imaging were consecutively selected for this single‐center retrospective observational study. Patient's demographic and clinical information was collected from the hospital's electronic medical records. Coronary angiograms and OCT images were compared via offline software.

**Results:**

A total of 390 patients were chosen as the final study population. HP was identified in 186 patients (47.7%) and was relatively less in cases of unstable angina pectoris (UAP) than in stable angina pectoris (SAP) (89/233 [38.2%] vs. 97/157[61.8%]). The HP group had greater prevalence rates of previous myocardial infarction and SAP and higher levels of triglycerides and uremia (median, 1.67 vs. 1.31 mmol/L [*p* = .01] and 364.22 ± 91.80 vs. 341.53 ± 77.64 µmol/L [*p* = .01], respectively). Using multivariate analysis, SAP and long lesion length were shown to be stand‐alone indicators of HP. HP presented with more severe stenosis as well as a longer lesion length and had more vulnerable and more complex features. In HP lesions, UAP patients had more plaque ruptures and thrombosis, whereas SAP patients had lower uric acid levels and more multiple HPs(≥3 HPs).

**Conclusion:**

Clinical presentation of SAP and long lesion length were strong predictors for HP in patients with AP. Patients with HP presented with more severe stenosis, longer lesion lengths, greater inflammation, and vulnerability.

## 1 INTRODUCTION

Angina pectoris (AP), especially refractory or persistent angina, is common in ischemic cardiomyopathy patients and distinctly correlated with an enhanced risk of prolonged severe adverse cardiologic events or rehospitalization.^1^, ^2^ However, the mechanism for this is currently unclear. Researchers have focused mostly on these plaques' instability during the past 30 years, and recurrent attacks of AP or the risk of acute myocardial infarction (AMI) or sudden death from AP remain difficult to predict. Pathology studies have suggested that healed plaques (HPs) may often be identifiable at nonculprit coronary locations of patients who die from sudden cardiac arrest or other causes.^3^, ^4^ Existing studies also contend that plaque healing may contribute to the natural progression of atherosclerotic disease and could explain the intermittent, rapid growth of plaques.^3^, ^4^

Recently, optical coherence tomography (OCT) imaging demonstrated enhanced sensitivity and specificity in detecting HP in vivo compared to histology as the gold standard.^5^ Other studies have demonstrated that HP presents with more severe luminal narrowing and more vulnerability to inflammation^6^, ^7^ and tried to reveal predictors of layered plaques.^8^, ^9^ However, the study included patients with AMI, which is usually associated with more vulnerable and inflammatory plaques. Several studies suggested that AMI patients presented with more multiple simultaneous plaque ruptures, compared with only a minority of those in patients with unstable angina pectoris (UAP) or stable angina pectoris (SAP).^10^, ^11^ Independent clinical predictors of multiple plaque ruptures were presented with AMI.^10^, ^11^ Whereas plaques with multiple ruptures might develop multiple healings. Few attempts have been made to example only patients with AP. Thus, we sought to elucidate the clinical and laboratory indicators of HP in AP patients. We also specifically focused on comparing the incidences and morphological properties of HP between SAP and UAP patients using in vivo OCT imaging.

## 2 METHODS

### 2.1 Study population

The present investigation was a single‐center retrospective observational study into which AP patients who received preintervention OCT assessment in Ward 12 of Anzhen Hospital in Beijing, China, between January 2013 and June 2018 were enrolled consecutively.

Stable AP was described as chest pain on exertion that had not changed in frequency, intensity, or duration of symptoms for ≥1 month and/or a positive stress test. UAP was defined by the presence of new or developing chest pain on exertion or rest within 2 weeks, in the absence of elevated cardiac enzymes (creatine kinase‐MB < 6.3 ng/ml and high‐sensitivity troponin I < 19.8 pg/ml, the threshold of cardiac enzymes of Anzhen Hospital).^12^

We collected patient demographic and clinical information, including basic vitals; age and sex; histories of smoking, hypertension, hyperlipidemia, and diabetes mellitus; family history of heart disease; medical history; and laboratory data at admission from the hospital electronic medical record. Percutaneous coronary intervention procedure data and OCT images were also collected. A diagnosis of prior myocardial infarction (MI) was recorded, according to medical documentation, pathological Q‐waves on electrocardiography with regional wall motion abnormalities, and/or presence of localized nonviable myocardium.

Among 1089 patients with available OCT images, patients with stent‐based events or postinterventional imaging records only (*n*= 368) were excluded; 82 patients with low‐quality OCT images caused by artifacts in the blood or too short a pullback were also excluded, and another 48 were excluded because of large thrombi or serious calcifications that impeded plaque visualization of the underlying layer. Those coronary heart disease etiology involving spontaneous coronary artery dissection, spasm, or myocardial bridge (*n* = 41) were also eliminated. Finally, patients with incomplete data (*n* = 82) and AMI (*n* = 78) were excluded from the final analysis. Thus, the final analysis was done on 390 patients (Supporting Information: Figure S1). For all enrolled patients in this study, the OCT was performed on a native “de novo” atherosclerotic vessel which was considered to be the culprit lesion.

All procedures complied with the Declaration of Helsinki, and our work received ethical approval from the Beijing Anzhen Hospital, Capital Medical University. Since we extracted our data retrospectively from patient medical records, the need to obtain documented informed consent was waived.

### 2.2 Coronary angiography assessment

Two experienced investigators analyzed coronary angiograms using offline software (Xcelera; Philips). We counted the amount of epicardial coronary arteries carrying stenotic lesions (diameter stenosis [DS] ≥ 50%). The term “eccentric lesions” refers to lesions that have one luminal edge outside the one‐fourth of the apparent vessel lumen. The same criteria were used for concentric lesions as long as they involved both luminal borders. To confirm the classification of lesions, multiple angiographic angles were used whenever possible. When the vessel was visible on moving images during the heart cycle in the absence of contrast injection, the presence of calcification was noted.^13^ Multivessel disease was described as having ≥2 coronary arteries or left main disease with ≥50% luminal narrowing. Lesions were stratified as Class A, B, or C according to angiographic assessment (e.g., longitudinal length, eccentricity, and tortuosity) based on the American Heart Association/American College of Cardiology stratification, and Class B2/C lesions (lesions with either two manifestations of Class B or one manifestation of Class C) were regarded as complex. We matched the angiography and OCT images with the help of landmarks like side branches and calcifications. The following measurements were taken: reference diameter, minimum lumen diameter, DS, and lesion length. Stenoses with a diameter of >70% were regarded as severe.^14^ The culprit plaque was evaluated by cardiologists who performed the angiography via coronary angiography findings, electrocardiography, and echocardiography. In the case of multiple stenosis patients, the plaque with the narrowest segment or with acute thrombus was considered to be the culprit lesion.^7^ Our data collection included information on the culprit plaque, and, in each case, the culprit plaque was evaluated offline and verified by two trained evaluators at Anzhen Hospital.

### 2.3 OCT image collection and analysis

OCT imaging was conducted with a frequency‐domain OCT system (C7‐XR OCT intravascular imaging system; LightLab). Intracoronary OCT imaging was performed following standard procedures. All collected OCT images were analyzed via an offline review workstation (IlumienOptis; St Jude Medical) by two separate researchers blinded to the study participants' demographic, clinical, and angiographic information. Any disagreements were resolved by consulting a third reviewer to reach a consensus. According to current consensus criteria for OCT imaging,^15^ fibrous plaques have a relatively homogeneous and high‐backscattering signal and lipid plaques constitute a signal‐poor area with diffuse borders, respectively. Lipid‐rich plaques (LRPs) were described as having a lipid arc of >90° and a lipid length of >1 mm. For each lipid plaque segment examined by OCT imaging, the thinnest fibrous cap thickness, as well as the maximal lipid arc, was recorded. A thin‐cap fibroatheroma (TCFA) was considered an LRP with the thinnest part of the fibrous cap being <65 µm.

**Figure 1:**
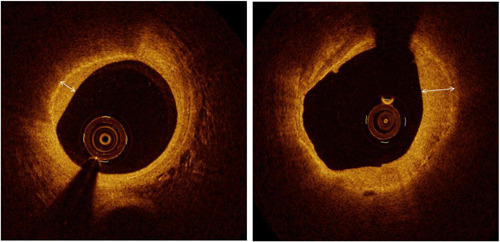
Optical coherence tomography images of healed plaques. Healed plaque, appears as a heterogeneous, signal‐rich layer of tissue (arrowhead) overlying a lipid‐rich plaque.

The lipid index was calculated as the product of the average lipid arc and lipid length. Calcification plaques (CPs) were recorded as areas with heterogeneous or low signal attenuation and sharp borders. Macrophage accumulations were defined by the existence of highly backscattering focal granular areas in the fibrous cap. Microvessels were identified by the recognition of small, signal‐poor vesicular or tubular structures in ≥3 contiguous frames. Cholesterol crystals were described as thin and linear regions with high signal intensity in plaques, without backscattering. Thrombus was described by a protrusion of ≥250 mm within the vessel lumen with signal attenuation. Plaque rupture was described by the discontinuity of a fibrous cap and cavity development within the plaque.^16^, ^17^ HP were described as those with ≥1 layer(s) with varying optical densities and an obvious demarcation from underlying tissues on OCT (Figure 1) as described in prior OCT research^3^, ^7^, ^18^ and a current histology verification study.^5^ Multiple HPs were defined as ≥3 HPs in the culprit lesion. For two separate plaques to exist in the same vessel, the length of the intervening reference segment had to be >5 mm; if not, visually distinct plaques were instead regarded as a single, elongated plaque. We measured the minimal lumen area (LA) of each lesion. The reference LA was estimated as the mean area of the largest lumen proximal or distal to the stenosis, within the same segment. The percentage of LA stenosis was computed as follows: ([reference LA − minimal LA]/reference LA) × 100.^19^

Inter‐ and intraobserver agreements were evaluated via *κ* coefficient statistics. The intraobserver variability was calculated in 80 randomly selected plaques 2 weeks apart. The inter‐variability was assessed by two independent physicians at two separate time points. The intra‐ and interobserver *κ* coefficients were 0.89 and 0.86 for HP, 0.96 and 0.93 for LRPs, 0.95 and 0.94 for CP, and 0.87 and 0.82 for macrophage infiltration, respectively.

### 2.4 Statistical analysis

Continuous variables, with normal distribution, are presented as mean ± standard deviation values and those with skewed distribution are presented as median (25th–75th percentiles). We compared continuous data using independent‐samples *t* or Mann–Whitney *U* test. Categorical data are shown as frequency (percentage) values and were analyzed via the chi‐squared or Fisher's exact test. Established cardiovascular disease risk factors (including clinical presentation, age, sex, prior MI, hypertension, diabetes mellitus, and hyperlipidemia) angiographic findings, and variables with *p* < .10, based on univariate analysis were further analyzed via multivariate analysis.

All data analyses employed the R version 3.3.2 software (http://www.R-project.org; The R Foundation for Statistical Computing) and a free statistics analysis platform. A two‐tailed *p* < .05 was set as the significance threshold.

## 3 RESULTS

### 3.1 Frequency of HP

Out of the 390 AP patients, culprit HP was detected in 186 patients (47.7%). Healed coronary plaques were less frequently observed in patients with UAP than SAP (89/233 [38.2%] vs. 97/157 [61.8%]) (Table 1).

**Table 1:** Baseline and angiographic characteristics of the patients

Variables	All (*n* = 390)		*p*‐Value	SAP (*n* = 157)		*p*‐Value	UAP (*n* = 233)		*p*‐Value	*p*′‐Value^a,b^
	Patients without HP (*n* = 204, 52.3%)	Patients with HP (*n* = 186, 47.7%)		Patients without HP (*n* = 6038.2%)	Patients with HP^a^ (*n* = 9761.8%)		Patients without HP (*n* = 144, 61.8%)	Patients with HP^b^ (*n* = 8938.2%)		
*Clinical presentation*			<.001							
SAP	60 (29.41%)	97 (52.15%)								
UAP	144 (70.59%)	89 (47.85%)								
Age, years	59.61 ± 12.17	59.53 ± 11.10	.948	58.6 ± 11.7	60.0 ± 12.0	.484	60.0 ± 12.4	59.0 ± 10.0	.528	.561
Male	147 (72.06%)	146 (78.49%)	.142	42 (70%)	75 (77.3%)	.404	105 (72.9%)	71 (79.8%)	.305	.819
Previous MI	21 (10.34%)	33 (17.93%)	.031	8 (13.3%)	20 (20.8%)	.33	13 (9.1%)	13 (14.8%)	.266	.38
Previous PCI	31 (15.27%)	35 (19.02%)	.327	9 (15%)	22 (22.9%)	.318	22 (15.4%)	13 (14.8%)	1	.223
Hypertension	104 (50.98%)	105 (56.76%)	.254	31 (51.7%)	52 (53.6%)	.942	73 (50.7%)	53 (60.2%)	.201	.448
Diabetes mellitus	51 (25.25%)	56 (30.27%)	.27	13 (21.7%)	28 (28.9%)	.417	38 (26.8%)	28 (31.8%)	.5	.782
Hyperlipidemia	93 (45.81%)	97 (52.43%)	.193	22 (36.7%)	49 (50.5%)	.126	71 (49.7%)	48 (54.5%)	.557	.689
Hyperuricemia	5 (2.46%)	7 (3.80%)	.447	2 (3.3%)	3 (3.1%)	1	3 (2.1%)	4 (4.5%)	.432	.711
Peripheral atherosclerosis	11 (5.39%)	18 (9.78%)	.101	4 (6.7%)	11 (11.5%)	.479	7 (4.9%)	7 (8%)	.499	.582
AF	3 (1.48%)	3 (1.64%)	.898	1 (1.7%)	1 (1.1%)	1	2 (1.4%)	2 (2.3%)	.637	.609
Renal insufficiency	2 (0.99%)	2 (1.09%)	.921	0 (0%)	2 (2.1%)	.524	2 (1.4%)	0 (0%)	.526	.498
*Smoking*			.544			.734			.119	.076
None	104 (51.49%)	90 (48.91%)		28 (46.7%)	51 (53.1%)		76 (53.5%)	39 (44.3%)		
Past	25 (12.38%)	30 (16.30%)		7 (11.7%)	10 (10.4%)		18 (12.7%)	20 (22.7%)		
Current	73 (36.14%)	64 (34.78%)		25 (41.7%)	35 (36.5%)		48 (33.8%)	29 (33%)		
Family history	36 (17.73%)	39 (21.20%)	.39	11 (18.3%)	21 (21.9%)	.742	25 (17.5%)	18 (20.5%)	.697	.956
*Medications history at admission*										
Statin at admission	115 (56.65%)	113 (61.08%)	.376	35 (58.3%)	59 (60.8%)	.887	80 (55.95)	54 (61.4%)	.501	1
Aspirin	117 (57.92%)	114 (61.62%)	.458	32 (54.2%)	61 (62.9%)	.368	85 (59.4%)	53 (60.2%)	1	1
P_2_Y_12_ inhibitor	58 (28.71%)	68 (36.76%)	.092	16 (27.1%)	38 (39.2%)	.173	42 (29.4%)	30 (34.1%)	.545	.826
ACEI/ARB	32 (15.84%)	39 (21.20%)	.175	7 (11.9%)	19 (19.6%)	.301	25 (17.5%)	20 (23%)	.396	.702
Beta‐blocker	52 (25.87%)	44 (24.18%)	.702	15 (25.4%)	27 (28.1%)	.856	37 (26.1%)	17 (19.8%)	.357	.254
*Laboratory data*										
WBC, 10^9^/L	6.46 ± 1.71	6.54 ± 1.71	.634	6.7 ± 1.6	6.5 ± 1.9	.616	6.4 ± 1.8	6.5 ± 1.5	.421	.946
Hemoglobin, g/L	152.34 ± 31.82	154.98 ± 31.00	.414	154.7 ± 33.0	153.1 ± 30.9	.761	151.4 ± 31.4	157.1 ± 31.2	.184	.389
Platelet, 10^9^/L	213.18 ± 62.64	206.64 ± 54.11	.28	223.4 ± 76.0	206.0 ± 56.1	.105	208.9 ± 55.9	207.4 ± 52.2	.839	.859
LDL‐C, mmol/L	2.40 ± 1.13	2.58 ± 0.94	.108	2.6 ± 1.5	2.6 ± 0.9	.88	2.3 ± 0.9	2.6 ± 1.0	.099	.745
TC, mmol/L	4.08 ± 1.28	4.29 ± 1.16	.099	4.3 ± 1.6	4.3 ± 1.0	.901	4.0 ± 1.1	4.3 ± 1.3	.05	.731
Triglycerides, mmol/L	1.31 (0.96–1.92)	1.67 (1.16–2.24)	.01	1.6 ± 0.7	1.8 ± 1.1	.141	1.6 ± 1.1	2.3 ± 2.8	.013	.162
HDL‐C, mmol/L	1.08 ± 0.25	1.02 ± 0.37	.067	1.1 ± 0.3	1.0 ± 0.2	.043	1.1 ± 0.2	1.0 ± 0.5	.321	.954
Hcy, µmol/L	15.37 ± 9.54	14.20 ± 8.04	.215	14.6 ± 9.6	13.8 ± 7.4	.584	15.7 ± 9.5	14.6 ± 8.7	.417	.525
Glu, mmol/L	6.18 ± 2.00	6.19 ± 1.93	.959	6.0 ± 1.7	6.2 ± 1.9	.605	6.3 ± 2.1	6.2 ± 1.9	.938	.774
Creatinine, µmol/L	71.49 ± 13.19	73.30 ± 13.33	.184	70.9 ± 10.6	72.7 ± 14.5	.416	71.7 ± 14.1	74.0 ± 12.0	.223	.535
UA, µmol/L	341.53 ± 77.64	364.22 ± 91.80	.01	330.3 ± 76.6	348.7 ± 92.4	.209	346.0 ± 77.9	381.6 ± 88.4	.002	.016
BNP, pg/ml	28.50 (17.00‐54.75)	30.00 (18.00‐49.00)	.579	25.0 (14.0, 46.0)	34.0 (20.0, 63.0)	.38	30.0 (17.5, 59.5)	28.0 (17.2, 36.2)	.388	.337
PT, s	10.91 ± 1.30	10.83 ± 0.83	.487	10.7 ± 0.6	10.8 ± 0.9	.484	11.0 ± 1.5	10.9 ± 0.8	.558	.234
INR	0.98 ± 0.12	0.97 ± 0.08	.409	1.0 ± 0.1	1.0 ± 0.1	.67	1.0 ± 0.1	1.0 ± 0.1	.568	.163
APTT, s	31.86 ± 2.97	31.56 ± 3.23	.358	31.8 ± 3.2	31.9 ± 3.0	.854	31.9 ± 2.9	31.2 ± 3.4	.114	.151
FBG, mg/dl	3.05 ± 0.63	3.11 ± 0.64	.356	3.1 ± 0.5	3.0 ± 0.7	.735	3.0 ± 0.7	3.2 ± 0.6	.087	.082
d‐d, ng/ml	68.00 (42.00–112.00)	70.00 (40.00–113.00)	.503	84.6 ± 58.9	82.6 ± 62.0	.846	93.0 ± 109.6	87.0 ± 64.2	.651	.640
FDP, µg/ml	1.43 ± 1.34	1.30 ± 0.95	.288	1.2 ± 0.9	1.3 ± 0.9	.493	1.5 ± 1.5	1.3 ± 1.0	.199	.873
hsTNI, µg/L	0.00 (0.00–0.02)	0.01 (0.00–0.04)	.453	0.0 ± 0.1	0.1 ± 0.2	.276	0.1 ± 0.2	0.1 ± 0.2	.637	.637
NEU, 10^9^/L	4.02 ± 1.35	4.06 ± 1.39	.81	4.2 ± 1.3	4.1 ± 1.5	.569	3.9 ± 1.4	4.0 ± 1.2	.611	.874
hsCRP, mg/L	0.90 (0.41–2.29)	1.20 (0.54–2.69)	.764	2.3 ± 4.0	2.3 ± 3.8	.891	2.5 ± 4.2	2.7 ± 3.7	.646	.516
GA, %	15.00 ± 3.76	14.74 ± 2.97	.477	14.7 ± 2.8	14.7 ± 3.0	.996	15.1 ± 4.1	14.8 ± 2.9	.538	.782
*Angiographic characteristics*										
OCT vessal			.213			.072			.821	.372
LAD	130 (63.73%)	134 (72.04%)		36 (60%)	74 (76.3%)		94 (65.3%)	60 (67.4%)		
LCX	29 (14.22%)	21 (11.29%)		13 (21.7%)	10 (10.3%)		16 (11.1%)	11 (12.4%)		
RCA	45 (22.06%)	31 (16.67%)		11 (18.3%)	13 (13.4%)		34 (23.6%)	18 (20.2%)		
Lesion length	13.56 ± 5.18	19.40 ± 6.37	<.001	14.0 ± 5.1	19.3 ± 6.4	<.001	13.4 ± 5.2	19.5 ± 6.4	<.001	.812
MLD, mm	1.58 ± 0.57	1.38 ± 0.45	<.001	1.5 ± 0.5	1.4 ± 0.5	.208	1.6 ± 0.6	1.4 ± 0.5	.001	.812
RVD, mm	3.82 ± 0.63	3.87 ± 0.55	.586	3.7 ± 0.6	3.9 ± 0.5	.155	3.9 ± 0.6	3.9 ± 0.6	.76	.682
DS, %	58.76 ± 11.77	64.33 ± 10.39	<.001	60.4 ± 10.5	64.5 ± 9.6	.013	58.1 ± 12.2	64.1 ± 11.2	<.001	.781
Multivessel disease	49 (24.02%)	71 (38.17%)	.002	17 (28.3%)	38 (39.2%)	.226	32 (22.2%)	33 (37.1%)	.021	.886
Type B2 + Type C	60 (29.41%)	96 (51.61%)	<.001	19 (31.7%)	50 (51.5%)	.023	41 (28.5%)	46 (51.7%)	<.001	1
Eccentric lesion	95 (46.6%)	107 (57.5%)	.039	31 (51.7%)	56 (57.7%)	.563	64 (44.4%)	51 (57.3%)	.563	1
Calcified lesion	48 (23.5%)	74 (39.8%)	<.001	18 (30%)	40 (41.2%)	.212	31 (51.7%)	56 (57.7%)	.563	.785

*Note*: Values are shown as *n* (%), mean ± standard deviation, or median (25th–75th percentile).

Abbreviations: ACEI, angiotensin‐converting enzyme inhibitor; AF, atrial fibrillation; APTT, activated partial thromboplastin time; ARB, angiotensin II receptor blocker; DS, diameter stenosis; FBG, fibrinogen; FDP, fibrin degradation product; GA, glycated albumin; Glu, glucose; HDL‐C, high‐density lipoprotein cholesterol; HP, healed plaque; hsTNI, high‐sensitivity troponin I; hs‐CRP, high‐sensitive C‐reactive protein; INR, international normalized ratio; LAD, left anterior descending artery; LCX, left circumflex artery; LDL‐C, low‐density lipoprotein cholesterol; MI, myocardial infarction; MLD, minimal lumen diameter; NEU, neutrophilic; PCI, percutaneous coronary intervention; PT, prothrombin time; RCA, right coronary artery; RVD, reference vessel diameter; SAP, stable angina pectoris; TC, total cholesterol; UA, uremia; UAP, unstable angina pectoris; WBC, white blood cell.

^a,b^ *p*′‐value refers to the comparison of HP lesions between UAP and SAP patients.

### 3.2 Characteristics of AP patients with culprit HP

**Figure 2:**
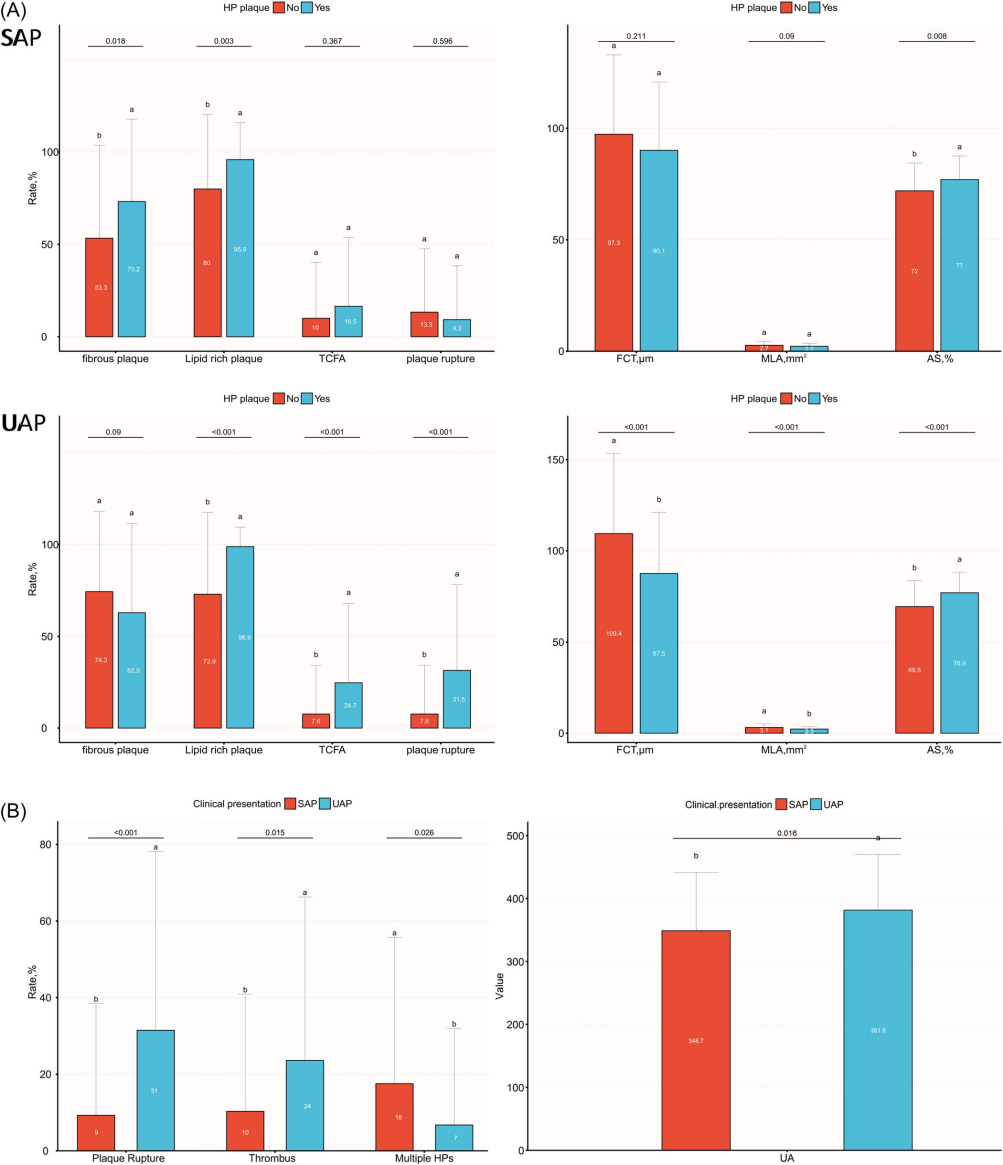
Characteristics between SAP and UAP patients. (A) OCT morphological characteristics are based on the culprit plaque phenotype (healed and nonhealed) in SAP and UAP patients. (B) Characteristics in HP lesions between SAP and UAP patients. AS, area stenosis; FCT, fibrous cap thickness; HP, healed plaque; MLA, minimal lumen area; OCT, optical coherence tomography; SAP, stable angina pectoris; TCFA, thin cap fibroatheroma; UAP, unstable angina pectoris. Letters a and b show statistically significant differences between variables.

Table 1 shows the baseline clinical characteristics. Overall, compared to the group without HP, those with HP exhibited an enhanced incidence of prior MI (17.93% vs. 10.34%; *p* = .031), and their levels of triglycerides and uremia were significantly higher (median, 1.67 vs. 1.31 mmol/L [*p* = .01] and 364.22 ± 91.80 vs. 341.53 ± 77.64 µmol/L [*p* = .01], respectively). The same laboratory trends could also be seen among UAP patients. In the SAP population, no obvious differences were seen in clinical manifestation between the two patient populations, aside from the level of high‐density lipoprotein cholesterol (HDL‐C) (1.1 ± 0.3 vs. 1.0 ± 0.2 µmol/L; *p* = .043). HP patients in SAP had lower uric acid levels than those in UAP (348.7 ± 92.4 vs. 381.6 ± 88.4 µmol/L, *p* = .016) (Figure 2B). No else difference was found between the two groups in clinical characteristics (Table 1).

### 3.3 Angiographic findings of HP in AP patients

Quantitative coronary analysis findings are detailed in Table 1. HP patients exhibited longer lesion lengths, a greater degree of DS, and more complex lesions (Type B2/C). Smaller minimal lumen diameters and more multivessel lesions could be observed in UAP patients with HP. Eccentric and calcified lesions were more common in patients with HP than without HP among the total study population. However, no significant differences were found between the subgroups analyzed. Moreover, multiple HPs were related to long lesion length (Supporting Information: Figure S2 and Table S2).

### 3.4 Predictors of HP

Univariate analysis showed that SAP, prior MI, lesion length, DS%, type B2/C lesions, triglycerides, and uremia were significant predictors of HP (Supporting Information: Table S1). In the multivariable analysis, SAP and lesion length were independent predictors for patients presenting with HP (Table 2).

**Table 2:** Multivariate regression analysis predictors for culprit healed plaque

	Odds ratio	95% CI	*p*‐Value
Clinical presentation			
SAP	1.00		
UAP	0.35	0.21–0.59	<.0001
Male	0.71	0.36–1.39	.315
Age, years	1.01	0.99–1.04	.252
Prior myocardial infarction	1.52	0.72–3.22	.273
Hypertension	1.15	0.68–1.94	.6
Diabetes mellitus	1.09	0.61–1.94	.767
Hyperlipidemia	1.36	0.81–2.28	.25
MLD, mm	2.21	0.75–6.55	.151
DS, %	1.05	1.00–1.11	.05
Multivessel disease	1.16	0.62–2.19	.646
Type B2 + Type C	1.63	0.91–2.94	.101
Lesion length, mm	1.18	1.12–1.24	<.0001
LDL‐C, mmol/L	1.19	0.91–1.56	.215
TG, mmol/L	1.12	0.91–1.38	.296
HDL‐C, mmol/L	0.52	0.24–1.16	.112
UA, µmol/L	1.00	1.00–1.01	.056

Abbreviations: CI, confidence interval; DS, diameter stenosis; HDL‐C, high‐density lipoprotein cholesterol; LDL‐C, low‐density lipoprotein cholesterol; MLD, minimal lumen diameter; SAP, stable angina pectoris; TG, triglyceride; UA, uremia; UAP, unstable angina pectoris.

### 3.5 Comparison of the morphological characteristics between SAP and UAP patients

In SAP patients, OCT analysis showed that HP correlated with greater prevalence rates of fibrous plaques, LRPs, calcification, macrophage accumulation, and more severe area stenosis (AS) as well as a longer lipid core length. Among UAP patients, HP had more vulnerable and more complex features, demonstrated by higher rates of LRPs, thin cap fibroatheroma (TCFA), cholesterol crystals, macrophage accumulation, plaque rupture, thrombus, and thinner fibrous cap thickness as well as a more severe lipid index, max lipid arc, minimal lumen area, and AS (Table 3 and Figure 2A). HP patients in UAP had more plaque ruptures and thrombosis than in SAP (31.5% vs. 9.3% [*p* < .001] and 23.6% vs. 10.3% [*p* = .026], respectively). Whereas SAP patients had more multiple HPs (≥3 HPs), compared with UAP patients (17.5% vs. 6.7%, *p* = .045) (Table 3 and Figure 2B).

**Table 3:** Morphological characteristics of the patients

Variables	SAP (*n* = 157)		*p*‐Value	UAP (*n* = 233)		*p*‐Value	*p*′‐Value^a,b^
	Patients without HP (*n* = 60)	Patients with HP^a^ (*n* = 97)		Patients without HP (*n* = 144)	Patients with HP^b^ (*n* = 89)		
Plaque type							
fibrous plaque	32 (53.3%)	71 (73.2%)	.018	107 (74.3%)	56 (62.9%)	.09	.178
Lipid‐rich plaque	48 (80%)	93 (95.9%)	.003	105 (72.9%)	88 (98.9%)	<.001	.371
TCFA, %	6 (10%)	16 (16.5%)	.367	11 (7.6%)	22 (24.7%)	<.001	.227
FCT, μm	97.3 ± 35.6	90.1 ± 30.6	.211	109.4 ± 44.0	87.5 ± 33.5	<.001	.582
Lipid length, mm	6.9 ± 3.4	8.9 ± 3.2	.001	6.3 ± 2.7	8.7 ± 3.4	<.001	.72
Mean lipid arc, °	185.2 ± 43.2	193.1 ± 53.4	.374	188.4 ± 48.2	204.9 ± 60.5	0.035	.169
Lipid index, mm°	1371.9 ± 968.6	1736.4 ± 869.4	.024	1226.7 ± 710.3	1854.3 ± 1060.0	<.001	.415
Max lipid arc, °	230.8 ± 61.6	246.0 ± 74.6	.225	230.1 ± 69.1	264.1 ± 80.1	.002	.118
Calcification	29 (48.3%)	69 (71.1%)	.007	59 (41.0%)	54 (60.7%)	.005	
Numbers of HPs							.045
1–2		80 (82.5%)			83 (93.3%)		
≥3		17 (17.5%)			6 (6.7%)		
Cholesterol crystals	13 (21.7%)	34 (35.1%)	.11	12 (8.3%)	33 (37.1%)	<.001	.893
Macrophage accumulation	21 (35.0%)	60 (61.9%)	.002	47 (32.6%)	66 (74.2%)	<.001	.102
Microvessels	17 (28.3%)	35 (36.1%)	.408	46 (31.9%)	38 (42.7%)	.128	.440
Thrombus	4 (6.7%)	10 (10.3%)	.624	13 (9.0%)	21 (23.6%)	.004	.026
plaque rupture	8 (13.3%)	9 (9.3%)	.596	11 (7.6%)	28 (31.5%)	<.001	<.001
MLA, mm^2^	2.7 ± 1.7	2.2 ± 1.4	.09	3.1 ± 2.0	2.3 ± 1.3	<.001	.869
RLA, mm^2^	9.3 ± 3.4	9.6 ± 2.8	.612	9.9 ± 3.2	9.9 ± 2.8	.9	.468
AS, %	72.0 ± 12.4	77.0 ± 10.6	.008	69.5 ± 14.1	76.9 ± 11.3	<.001	.964

*Note*: Values are shown as *n* (%) and mean ± standard deviation.

Abbreviations: AS, area stenosis; FCT, fibrous cap thickness; HPs, healed plaques; MLA, minimal lumen area; RLA, reference lumen area; SAP, stable angina pectoris; TCFA, thin cap fibroatheroma; UAP, unstable angina pectoris.

^a,b^ *p*′‐value refers to the comparison of HP lesions between UAP and SAP patients.

## 4 DISCUSSION

This study demonstrated that in patients with AP: (1) HP was identified in 47.7% of AP patients, (2) SAP patients had a higher prevalence rate of HP and multiple HPs (≥3 HPs) than UAP patients, (3) the clinical presentation of SAP and lesion length on coronary angiography were predictors for HP in AP patients, (4) HP presented with longer lipid lengths and more severe luminal stenosis. Compared to those in SAP patients, HP in UAP patients had more vulnerable and more complex features and (5) HP patients in UAP had higher uric acid levels, more plaque ruptures, and thrombosis than in SAP.

### 4.1 Prevalence of HP

In the past decades, HP could only be observed by pathological examinations performed at autopsy.^3^, ^4^ With the rapid development of intracoronary imaging techniques; however, HP is now identified in vivo via OCT imaging.^5^ Here, we recorded an in vivo HP incidence rate of 47.7% at the culprit site in AP patients. Prior autopsy investigations have demonstrated enhanced HP incidence rates up to 73% with a stenosis diameter of >50%.^4^ One pathology study reported that healed ruptures were found in 61% of hearts.^3^ Meanwhile, the in vivo HP incidence rate at the culprit site was 29% in acute coronary syndrome (ACS) patients,^7^ and 40.3% of patients with AMI exhibited layered culprit plaques.^8^ A meta‐analysis concluded the HP prevalence among the general population is 40%,^20^ which is similar to the result of our analysis. This meta‐analysis also demonstrated that the HP incidence was 37% in ACS patients and 46% in SAP patients, respectively, but our study demonstrated an enhanced prevalence of HP in the SAP population (61.8%). Other studies have documented similarly high rates in SAP patients.^21^ The prevalence of HP in autopsy studies was the highest, maybe because the entire coronary trees were examined in these studies and the included patients had all died of sudden cardiac death or ischemic heart disease. Pathology analysis also offers higher accuracy and greater positivity. The variability in the prevalence of nonculprit HP may be due to whether or not all three coronary arteries were examined.

### 4.2 Clinical presentation and HP

Here, HP patients displayed an enhanced incidence of both hypertriglyceridemia and hyperuricemia. In SAP patients, the level of HDL‐C was lower in those with HP. In a separate investigation, ACS patients with HP showed enhanced incidence rates of hyperlipidemia and diabetes mellitus.^21^ AMI patients with HP demonstrated enhanced levels of low‐density lipoprotein cholesterol.^8^ These findings indicate two things. First, the influence of common coronary risk factors on the prevalence of HP varies among reports,^8^, ^9^ which may be due to differences in study populations. Our multivariable analysis also demonstrated that common coronary risk factors are not predictors of HP. Second, risk factors, such as hypertriglyceridemia, hyperuricemia, and lower HDL‐C levels, are associated with higher risks for thrombosis and adverse events.^22^, ^23^, ^24^ These cardiovascular risk factors are also associated with a greater incidence of HP; thus, these risk factors may contribute to plaque rupture, followed by plaque healing. It is to be noted that HP patients in SAP had lower uric acid levels than those in UAP. One study reported that higher serum uric acid levels had an impact on coronary plaque stability in patients with coronary artery disease.^25^ This result indicated that high uric acid levels might induce plaque instability, which, in turn, leads to ACS occurrence. Conversely, low levels of serum uric acid might be associated with small plaque rupture and healing, which in turn leads to SAP occurrence. whether the levels of serum uric acid are strongly correlated with plaque healing requires a larger sample size to confirm.

Importantly, MI was more prevalent among patients with HP, in line with other recent OCT studies.^6^, ^9^ This clinical presentation also did match the pathological features.^3^ Here, we found that HP appeared more frequently in SAP patients, relative to AP patients, which is in line with the results of other recent OCT research reporting that layered plaques were more common in SAP patients, relative to ACS or AMI patients. Most scholars currently believe that an HP status is attained via an equilibrium between thrombogenicity and antithrombotic properties. Our findings suggest that SAP is an independent predictor for plaque healing, maybe because, after healing following plaque rupture, the patient clinically presents with SAP, not ACS. So, SAP may be the result of plaque healing, not the reason.

### 4.3 Angiographic findings of HP in AP patients

HP patients displayed longer lesion lengths, elevated DS, and more complex lesions (Class B2/C). These tendencies were also more pronounced in UAP patients than SAP patients, and the same results could be observed in other studies including ACS patients.^7^, ^21^ Atherosclerotic plaques gradually increase to a certain degree and result in severe narrowing or occlusion of the affected artery. It has been assumed in the past that plaque progression is linear; however, later studies showed that the progression was instead a phasic result of plaque rupture and healing.^3^, ^4^ A histopathological study showed that type III collagen is synthesized in the initial healing phase, then is replaced by type I collagen in a later phase. Eventually, the plaque volume becomes larger in just a short period. Thus, HP is accompanied by more stenosis and increasingly complicated lesions. In UAP patients with more unstable plaques, more plaque rupture and healing might occur. Lesion length, a higher degree of DS, and more complex lesions (Class B2/C). Thus, more severe stenosis and more multivessel lesions could be observed in UAP patients with HP, relative to those without HP.

In this study, we also observed the impact of other types of B2/C lesions, such as eccentric and calcified lesions, on the HP. One study concluded that lesions with HP had more eccentric lumens after stent implantation.^26^ Another study found that calcium deposition was associated with plaque stability.^27^ Our study showed that eccentric and calcified lesions were more common in patients with HP than without HP among the total study population. A similar but nonsignificant trend was also seen between subgroups analyzed. The reason for this result maybe is the low number of subgroup participants.

### 4.4 Lesion length and HP

Here, HP patients exhibited longer lesion lengths, further luminal constriction, and more complicated vascular lesions; moreover, lesion length was an independent predictor of HP. Further analyses showed that multiple HPs are related to lesion length. Recent studies have suggested an association of an HP phenotype with lumen narrowing at the culprit and nonculprit locations, which was also confirmed by our study.^7^ We consider that, if recurrent plaque rupture and healing occur in the same location, the plaque volume will increase and correlate with more severe stenosis of the coronary artery. Conversely, if they occur at different locations of a single vessel, then a longer lesion length would result. In most cases, plaque rupture and healing occurred at different locations.

### 4.5 OCT characteristics of HP

Here, LRPs, macrophage accumulation, and CP were excessively common in AP patients with HP, and these results match those of previous studies.^6^, ^7^, ^9^, ^28^ We also observed the existence of more severe AS as well as longer lipid lengths in HP patients. This suggests that culprit lesions in patients with HP were more vulnerable and had a higher level of inflammation. Thus, we believe that many of the HP had not healed. Some scholars have described them as “inactive volcanoes” within the vascular system and, under certain conditions, may erupt again.^29^ This also suggested that HP was somewhat vulnerable. Among UAP patients, lesions with HP had a greater vulnerability and more complex features. This finding similarly indicated that healing was a temporary state and not true healing.

Our study showed that in HP lesions, UAP patients had more plaque ruptures and thrombosis Whereas SAP patients had more multiple HPs. Some studies found that there was a higher incidence of nonculprit plaque rupture among SAP patients (22%) than those with UAP (12%). However, plaque ruptures were less likely to be covered by thrombus in SAP patients than in UAP patients (21% vs. 33%).^11^ The results of these studies were similar to our analysis. So we speculated that small plaque ruptures were accompanied by multiple self‐healing process which was clinically presented with SAP. A significant portion of the plaque ruptures in SAP patients, whether or not in culprit lesion, maybe were actually silent plaque ruptures, whereas in UAP patients, the plaque showed acute destabilization more frequently.

However, how to keep the HP stable or avoid repeated plaque rupture and healing is not clear. Intensive antithrombotic therapy was demonstrated to be highly efficacious toward coronary plaque erosion healing. Furthermore, multiple reports validated the numerous benefits of potent lipid‐lowering drugs (e.g., high‐dose statins, proprotein convertase subtilisin–kexin type 9 inhibitors) on plaque stabilization. Anti‐inflammatory agents are known to lower the risk of cardiovascular events. Although the mechanisms behind these benefits remain unclear, an improvement in plaque‐healing capacity may encourage faster healing. Considering the widely used method involving mechanisms of plaque instability, we should perform more research to further understand the protective mechanisms that convert “poor healers” into “good healers.” Therefore, further prospective studies will be necessary to reveal novel mechanisms of plaque healing and identify more effective means for promoting the stability of plaque healing.

### 4.6 Limitations

Our work encountered certain limitations. First, our study design was retrospective, so inherent selection bias may have been inadvertently introduced. Second, nonculprit plaques were not evaluated using systematic 3‐vessel imaging. This is because this technology is unsuitable in real‐world clinical practice and may bring about undue risk factors. Third, although a prior histology validation study reported elevated OCT specificity in diagnosing HP, diagnosing HP using OCT can still be challenging. Fourth, only a limited number of inflammatory and coagulation indexes were assessed. As such, we cannot say that a definite relationship exists between plaque healing and the inflammation and coagulation systems. In light of this, future investigations must identify more biomarkers to assess the association between plaque rupture and healing to better elucidate related mechanisms and pathology.

## 5 CONCLUSION

The clinical presentation of SAP and lesion length appeared to be strong predictors for HP in patients with AP. Furthermore, OCT analysis showed that HP presented with greater inflammation and vulnerability, more severe AS, and higher rates of macrophage accumulation, LRPs, calcification, cholesterol crystals, plaque rupture, and TCFA.

## CONFLICT OF INTEREST

The authors declare no conflict of interest.

## Supporting information

Supplementary information.

## Data Availability

The data analyzed for this article are available from the corresponding author upon reasonable request.
