# Supplementary material for: Predictors and morphological properties of culprit healed plaques in patients with angina pectoris
Source: Clin Cardiol. 2022 Sep 7;45(12):1199–210. doi: 10.1002/clc.23896 (PMC9748754; doi:10.1002/clc.23896)
Supplement: Supplementary file 1 — Supplementary information. [file CLC-45-1199-s001.docx]

**Supporting Information:**

**Supplemental Figure S1.** **Study Flowchart**

**
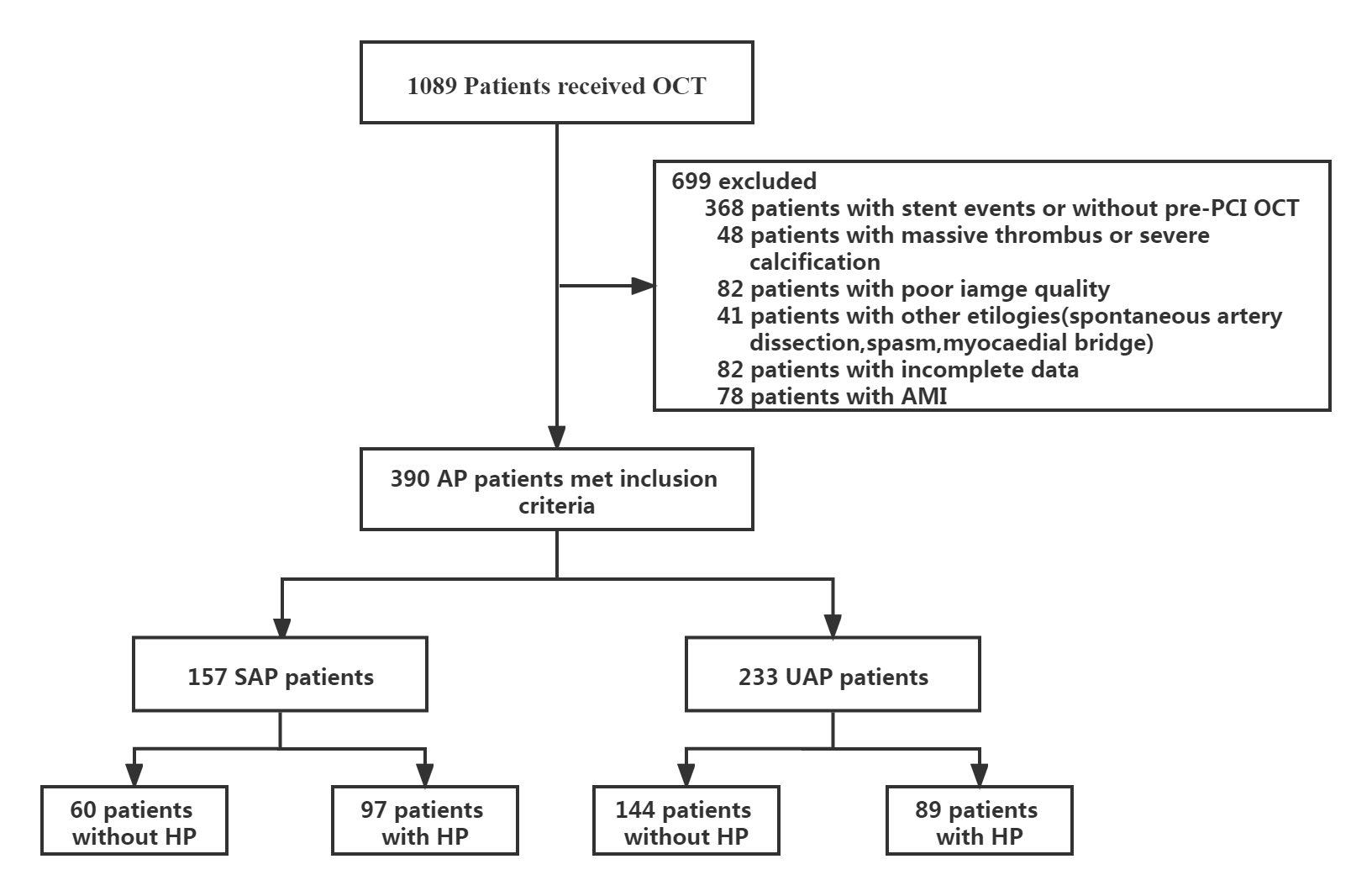
**

OCT, optical coherence tomography; PCI, percutaneous coronary intervention; AMI, acute myocardial infarction; AP, angina pectoris; SAP, stable angina pectoris; UAP, unstable angina pectoris.

**Supplemental Figue S2. The association of lesion length and incidence of multiple HPs**


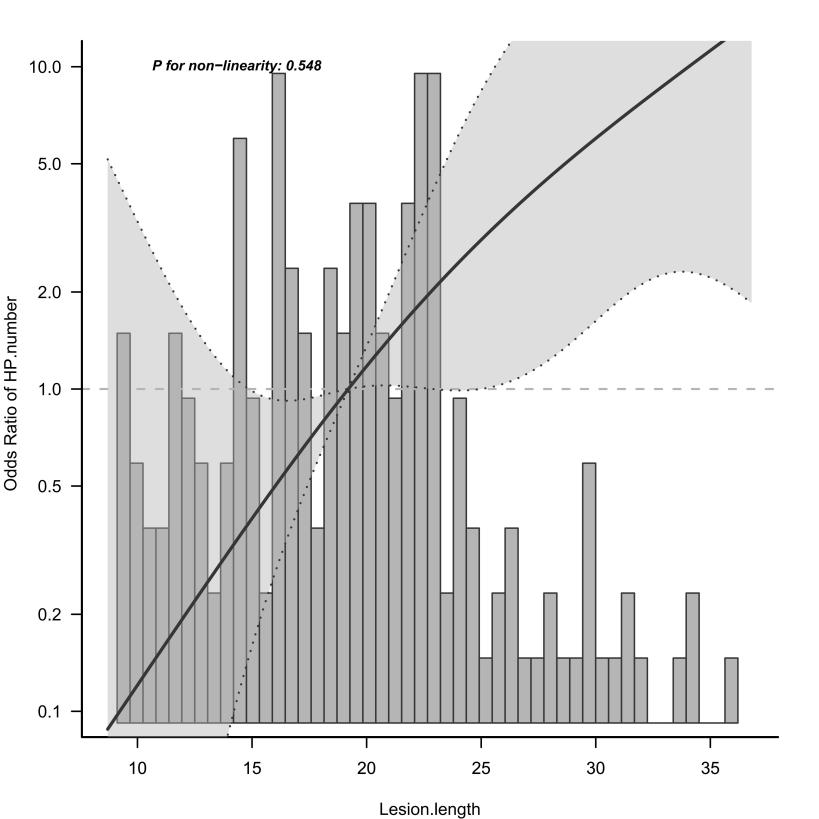


**Supplemental Table S1.** Univariate Analysis Predictors for culprit healed plaque

|  | Odds ratio | 95% CI | p value |
| --- | --- | --- | --- |
| Clinical presentation |  |  |  |
| SAP | 1.00 |  |  |
| UAP | 0.38 | 0.25- 0.58 | <0.0001 |
| Male | 1.42 | 0.89- 2.25 | 0.143 |
| Age, yrs | 1.00 | 0.98- 1.02 | 0.947 |
| Prior myocardial infarction | 1.89 | 1.05- 3.41 | 0.033 |
| Prior PCI | 1.30 | 0.77- 2.22 | 0.328 |
| Hypertension | 1.26 | 0.85- 1.88 | 0.254 |
| Diabetes mellitus | 1.29 | 0.82- 2.01 | 0.270 |
| Hyperlipidemia | 1.30 | 0.87- 1.94 | 0.193 |
| Hyperuricemia | 1.57 | 0.49- 5.02 | 0.451 |
| Peripheral atherosclerosis | 1.90 | 0.87- 4.14 | 0.105 |
| AF | 1.11 | 0.22- 5.58 | 0.898 |
| Renal insufficiency | 1.10 | 0.15- 7.92 | 0.921 |
| smoking |  |  |  |
| None | 1.00 |  |  |
| Past | 1.39 | 0.76- 2.53 | 0.287 |
| Current | 1.01 | 0.65- 1.57 | 0.954 |
| Family history | 1.25 | 0.75- 2.07 | 0.390 |
| Statin at admission | 1.20 | 0.80- 1.80 | 0.376 |
| warfarin | 0.00 | 0.00- Inf | 0.982 |
| Aspirin at admission | 1.17 | 0.78- 1.75 | 0.459 |
| P2Y12 inhibitor | 1.44 | 0.94- 2.21 | 0.092 |
| ACEI or ARB | 1.43 | 0.85- 2.40 | 0.176 |
| OCT vessal |  |  |  |
| LAD | 1.00 |  |  |
| LCX | 0.70 | 0.38- 1.29 | 0.258 |
| RCA | 0.67 | 0.40- 1.12 | 0.127 |
| Lesion length | 1.20 | 1.14- 1.25 | <0.0001 |
| MLD, mm | 0.46 | 0.30- 0.69 | 0.000 |
| RVD, mm | 1.15 | 0.82- 1.62 | 0.404 |
| DS, % | 1.05 | 1.03- 1.07 | <0.0001 |
| Multivessel disease | 1.95 | 1.26- 3.02 | 0.003 |
| Type B2+Type C | 2.56 | 1.69- 3.88 | <0.0001 |
| Beta blocker | 0.91 | 0.57- 1.45 | 0.702 |
| WBC | 1.03 | 0.91- 1.16 | 0.633 |
| Hb | 1.00 | 1.00- 1.01 | 0.415 |
| plat | 1.00 | 0.99- 1.00 | 0.280 |
| LDL-C | 1.18 | 0.96- 1.46 | 0.115 |
| TC | 1.16 | 0.97- 1.38 | 0.104 |
| TG | 1.30 | 1.06- 1.60 | 0.011 |
| HDL-C | 0.48 | 0.22- 1.08 | 0.075 |
| Hcy | 0.98 | 0.96- 1.01 | 0.218 |
| Glu | 1.00 | 0.90- 1.11 | 0.959 |
| Cr | 1.01 | 1.00- 1.03 | 0.184 |
| UA | 1.00 | 1.00- 1.01 | 0.010 |
| BNP | 1.00 | 1.00- 1.00 | 0.583 |
| PT | 0.93 | 0.77- 1.13 | 0.490 |
| PT% | 1.00 | 0.98- 1.02 | 0.995 |
| INR | 0.41 | 0.05- 3.50 | 0.414 |
| APTT | 0.97 | 0.91- 1.04 | 0.359 |
| FBG | 1.17 | 0.84- 1.62 | 0.356 |
| D-D | 1.00 | 1.00- 1.00 | 0.506 |
| FDP | 0.91 | 0.75- 1.09 | 0.292 |
| hsTN | 1.49 | 0.52- 4.21 | 0.456 |
| NEU | 1.02 | 0.87- 1.19 | 0.809 |
| hsCRP | 1.01 | 0.96- 1.06 | 0.764 |
| GA | 0.98 | 0.92- 1.04 | 0.477 |

Abbreviations:OR,odd ratio; CI, confidence interval;other abbreviations as same as Table 1.

**Supplemental Table S2** Logistic regression models evaluating the association between lesion length and incidence of multiple HPs

| Variable | n.total | n.HP% | Model 1 | | Model 2 | |
| --- | --- | --- | --- | --- | --- | --- |
|  |  |  | OR(95%CI) | P value | OR(95%CI) | P value |
| Lesion.length | 186 | 23 (12.4) | 1.18 (1.1~1.27) | <0.001 | 1.18 (1.09~1.27) | <0.001 |

Values in the table are OR (95% CI).

Abbreviations: HPs: healed plaques; Ref: reference.

Model 1, adjust for: none.

Model 2, adjusted for age and sex .
